# Engineering interfacial tissues: The myotendinous junction

**DOI:** 10.1063/5.0189221

**Published:** 2024-06-03

**Authors:** Finn Snow, Cathal O'Connell, Peiqi Yang, Magdalena Kita, Elena Pirogova, Richard J. Williams, Rob M. I. Kapsa, Anita Quigley

**Affiliations:** 1 Biomedical and Electrical Engineering, School of Engineering, RMIT University, Melbourne, VIC 3000, Australia; 2 Aikenhead Centre for Medical Discovery, St Vincent's Hospital, Fitzroy, VIC 3065, Australia; 3 School of Medicine, Deakin University, Geelong, Victoria, Australia; 4 Institute for Mental and Physical Health and Clinical Translation, School of Medicine, Deakin University, Geelong, Victoria, Australia; 5 Department of Clinical Neurosciences, St Vincent's Hospital Melbourne, Fitzroy, Melbourne, VIC 3065, Australia; 6 The Graeme Clark Institute, The University of Melbourne, Melbourne, VIC 3010, Australia; 7 Department of Medicine, The University of Melbourne, Fitzroy, Melbourne, VIC 3065, Australia

## Abstract

The myotendinous junction (MTJ) is the interface connecting skeletal muscle and tendon tissues. This specialized region represents the bridge that facilitates the transmission of contractile forces from muscle to tendon, and ultimately the skeletal system for the creation of movement. MTJs are, therefore, subject to high stress concentrations, rendering them susceptible to severe, life-altering injuries. Despite the scarcity of knowledge obtained from MTJ formation during embryogenesis, several attempts have been made to engineer this complex interfacial tissue. These attempts, however, fail to achieve the level of maturity and mechanical complexity required for *in vivo* transplantation. This review summarizes the strategies taken to engineer the MTJ, with an emphasis on how transitioning from static to mechanically inducive dynamic cultures may assist in achieving myotendinous maturity.

## INTRODUCTION

Musculoskeletal conditions continue to plague our community with approximately 1.71 × 10^9^ people affected worldwide, making them the leading contributor to disability globally.^1^ Although only being responsible for 2% of total hospital discharges, musculoskeletal conditions reported 5.4% of the total hospital costs for children and adolescents aged 20 years or younger, correlating to a $7.6 billion economic burden. In fact, in 2011, there was an estimated $213 billion annual cost of direct treatment and lost wages in the United States alone.^2^ Arising from various diseases, injuries, and myopathies, musculoskeletal conditions are largely incurable or irreparable. Trauma to muscles and tendon alone contributes 20.6% of all work-related musculoskeletal injuries in Australia (Safe Work Australia, 2016), in which the MTJ represents the primary site of injury at 58.7%.^3^ Current surgical methods, such as suturing, allografts, and xenografts, result in residual scar tissue that disrupts the fragile biomechanics of the muscle–tendon interface, and hence do not achieve the therapeutic rehabilitation required for patients to return to pre-injury activities. Although the interdisciplinary field of tissue engineering has previously encompassed interfacial tissues, this domain remains relatively unexplored, particularly regarding the MTJ. Groups that have targeted the MTJ have observed interface-specific markers indicating MTJ development; however, current efforts have failed to recapitulate the maturity and mechanical complexity of native MTJs. Herein, we review the finite studies targeted at MTJ regeneration, revealing a common trend that may be extrapolated to other interfacial tissues. In particular, this review focuses on the mechanosensitive traits of skeletal muscle and tendon constructs in three-dimensional (3D) culture, in which the prospect of maturing MTJ constructs under uniaxial strain has been explored.

To rationally design a MTJ engineering strategy, we should consider the process by which it is originally formed. Herein, we begin by describing the development and mechanical characteristics of MTJ constituents *in utero*, followed by the biomechanics of fully developed MTJs in humans. Subsequently, through summarizing the finite studies targeted at MTJ engineering, the lack of dynamic culture methods has been identified as a limitation. Finally, this gap has been linked back to the established literature on individual tissue responses to uniaxial strain in dynamic cultures, revealing a targeted future perspective aimed at recapitulating the native environment more appropriately to be identified.

## MTJ CHARACTERISTICS

### Embryonic development

MTJ formation is unequivocally reliant on the interactions between developing tissues during embryogenesis. Originating from the myotome, myogenesis relies on a complex, yet not fully elucidated signaling network comprised of molecules, such as Wingless-related integration site proteins (Wnts), sonic hedgehog (Shh), and bone morphogenetic proteins (BMPs).^4^ These molecules, especially Wnt1 and Wnt3a, heavily influence the expression of myogenic regulatory factors (MRFs) responsible for myogenic lineage progression and differentiation.^4,5^ Contrarily, tenogenesis originates from the undifferentiated mesenchymal cells within the lateral plate mesoderm.^6^ Initially, Shh signals derived from the notochord induce paired box transcription factors (Pax) 1 and 9, which regulate mesenchymal differentiation. This process ultimately commits SRY-box transcription factor 9 (Sox-9)-expressing chondrogenic mesenchymal cells to their tendinous fate.^7^ Post mesenchymal differentiation, tendon progenitors express various transcription factors, including Scleraxis (Scx), Mohawk (Mkx), early growth response 1 (Egr1), and early growth response 2 (Egr2).^8^

MTJ formation begins as tenocytes attach to skeletal muscle cells (myocytes). The chemical and mechanical signaling between these tissues during embryonic development is poorly understood; however, electron microscopy has been widely incorporated to analyze the origins of the MTJ. The earliest morphological modification observed at the MTJ is the formation of close associations between myogenic cells and tendon fibroblasts.^9^ Here, a dual identity can be seen as fibroblasts transdifferentiate by switching on a myogenic program, allowing fusion into myofibers.^10^ Simultaneously, extracellular material accumulates at the surface of muscle cells, representing the first appearance of the basement membrane.^9,11^ Myofibril production subsequently increases, allowing subsarcolemmal densities to appear at the intracellular surface of the MTJ.^9^ Finally, associations between myofibril thin filaments and subsarcolemmal densities occur, resulting in membrane folding, later depicted as invaginations.^11^

**FIG. 1.**
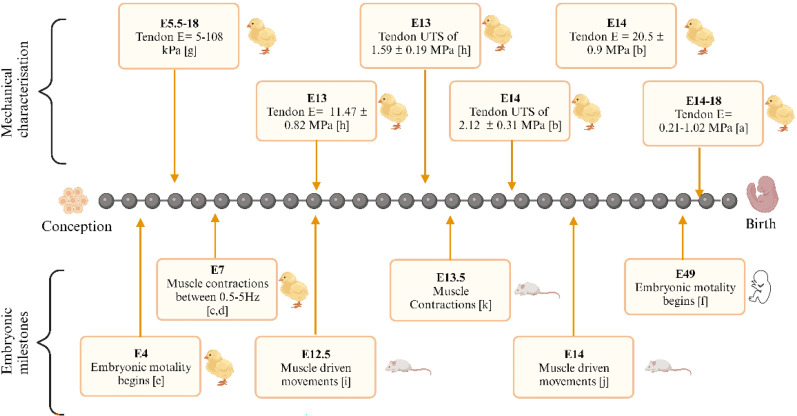
Timeline of embryonic milestones and mechanical properties from conception to birth. (a) Ref. 12, (b) Ref. 16, (c) Ref. 17, (d) Ref. 18, (e) Ref. 19, (f) Ref. 20, (g) Ref. 14, (h) Refs. 21 and 22, (i) Ref. 23, (j) Ref. 24, and (k) Ref. 25. Created with BioRender.com.

To date, there has been limited reported data regarding the mechanical properties of embryonic skeletal muscle, particularly in mammals. Although myotendinous development is a generic process in vertebrates, Fig. 1 displays not only the variability between species but also the rapid formation *in utero* as evident in the increased stiffness and ultimate tensile strength (UTS). A study by McBride *et al.* focused on individual tendons adjacent to the femur and tibiotarsus of fertilized white leghorn eggs. At post-fertilization day (PFD) 14, a Young's modulus (E), UTS, and strain at failure (SAF) were determined to be 0.216 ± 0.060 MPa, 2.052 ± 1.112 MPa, and 12.77% ± 1.91%, respectively. At PFD 17, these values had increased to 1.02 ± 0.27 MPa, 21.411 ± 2.634 MPa, and 29.83 ± 5.33, indicating not only the rapid pace of embryonic tendon development but also the disparity between embryonic and adult tendon mechanical properties.^12^ This disparity may be seen by Nakagaki *et al.*'s elastic modulus of caged, 8-month-old calcaneal tendon of chickens (210.51 ± 46.01 MPa).^13^ It has been determined from Marturano *et al.* that the elastic modulus of the embryonic tendon increased nonlinearly as a function of embryonic stage at both the nano- and microscale.^14^ A further in-depth review of embryonic development may be found here.^15^

### Composition

For the MTJ to develop into an integrated mechanical unit that can support and transmit great forces, many connective complexes must be considered.^26^ These connections must not only allow high tensile stresses to be endured but also act as the junction in which intracellular myofilament contractions transmit force to extracellular proteins found in the tendon.^27^ In total, five milestones of MTJ formation have been reported in the literature, including^26,28–31^

(i) Actin microfilaments extend from the last Z-line of sarcomeres and terminate in the ridge-like protrusions.
(ii) Actin-binding proteins cross bind highly aligned actin filaments together.
(iii) Actin filament bundles insert into the sarcolemma plasma membrane via an electron-dense subsarcolemmal layer. The anchorage of these actin filaments may be perpendicular or oblique.
(iv) Transmembrane proteins that link cytoskeletal elements to basal lamina components. Basil lamina is seemingly thicker in these regions.
(v) Proteins that link the basement membrane to collagen fibril-rich matrix in such a way that the collagen fibrils are parallel to the muscle myofilaments.

Two major and distinct transmembrane linkage systems have been described at the MTJ, which rely on the dystrophin-associated glycoproteins complex (DGC) and the binding and signaling protein “α7β1” integrin. Both systems constitute a structural link between cytoplasmic actin and tendinous extracellular matrix proteins. These links heavily rely on laminin 211, which is prevalent in both complexes, and represents the unique isoform that is found in adult human MTJs.^26,27^

### Ultrastructure

**FIG. 2.**
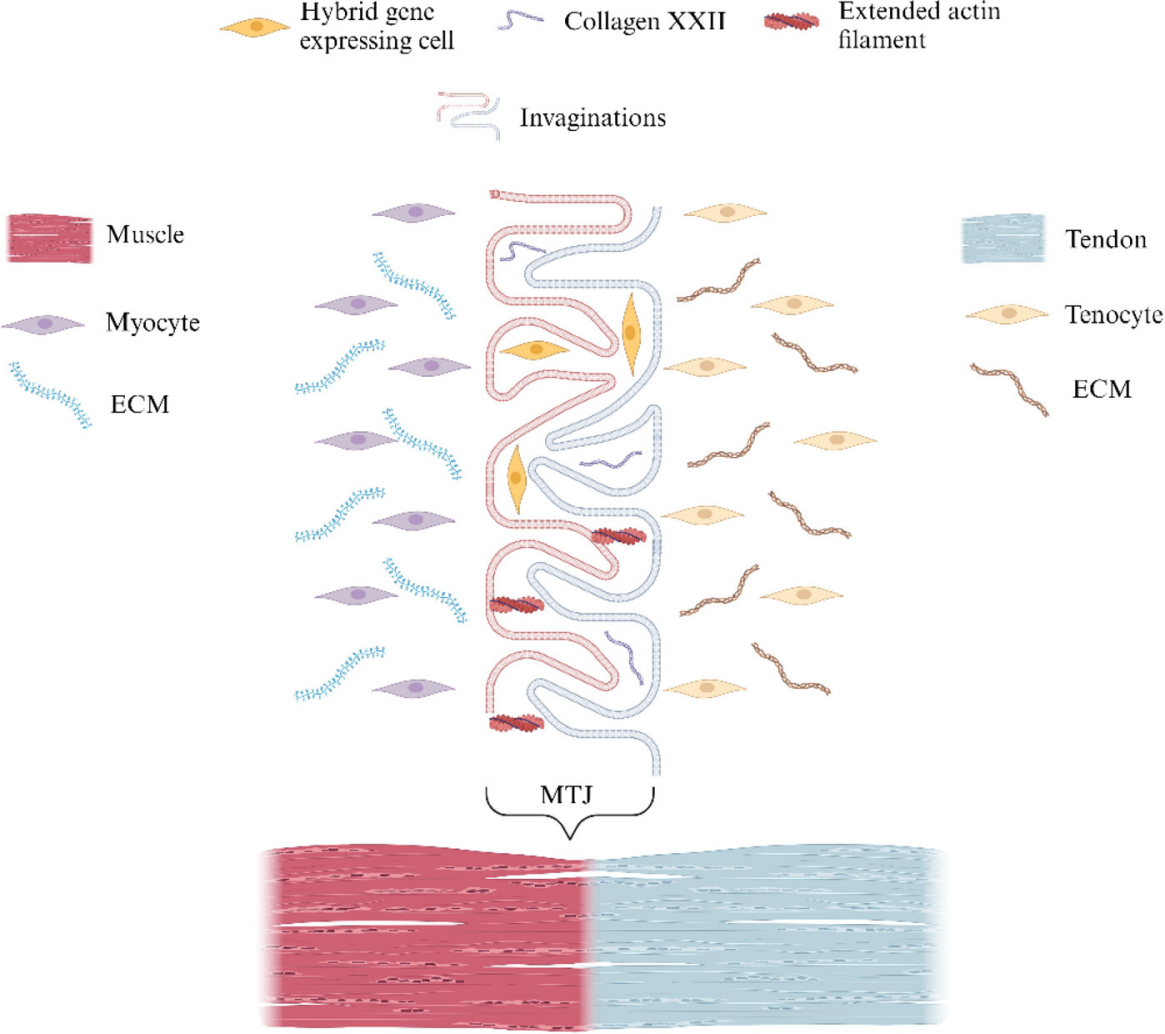
Ultrastructure of the MTJ. Created with BioRender.com.

The adult MTJ contains a complex morphology to combat stress concentrations and avoid injury. Advancements in scanning techniques, such as transmission and scanning electron microscopy (TEM and SEM, respectively), and focused ion beam (FIB) have steered scientists away from the idea of a simplistic planar divisional interface between skeletal muscles and tendons, revealing a more complex, interwoven design. Here, tendinous extracellular matrix (ECM) folds and protrudes into invaginations of the muscle cell membrane (sarcolemma) as represented in Fig. 2. In fact, while studying the human MTJ in 3D, Knudsen *et al.* more accurately described the connection between tissues as ridge-like protrusions^30^ that serve two primary mechanical purposes.

First, the interwoven nature of the MTJ greatly increases the contact area between skeletal muscles and tendons, therefore significantly reducing stress concentrations.^32^ 2D TEM images taken by Noonan *et al.* revealed a 10–20-fold increase in surface area compared to a smooth planar transition;^33^ however, recent 3D modeling by Knudsen *et al.* indicates an even greater increase.^30^ Second, the intertwining design positions the muscle cell membrane at acute angles relative to the applied force, compelling the sarcolemma to be predominately exposed to shear forces.^34^ Shear forces are optimal as cell membranes display greater strength of adhesion while subject to contractile shear stresses as opposed to tension loading.^35^ Thus, the ability of the MTJ membranes to transmit force is accentuated through invaginations which result in greater load-bearing capabilities. The ultrastructure of the MTJ may be seen below in Fig. 2.

### Biomechanics

Generally, collagen fibrils within the tendon are crimped until the onset of strain, resulting in an initial toe region of a stress–strain curve up until 2%–2.5% strain,^36^ in which tendonous strains primarily reside under physiological conditions.^37^ Once stretched beyond 4%, plastic deformation occurs, representing mild injury. If stretched over 10%, however, complete rupture will occur.^38,39^ The tendon is also described as a viscoelastic tissue, meaning compliance at low strain rates and resilience at high strain rates. This was elegantly displayed by Wren *et al.*, who increased the rate of strain from 1 to 10 mm/s during tensile loading of the human Achilles tendon (AT).^40^ Here, a greater strain rate resulted in increased E, UTS, and SAF from 816 ± 218 to 822 ± 211 MPa, 71 ± 17 to 86 ± 24 MPa, and 7.5% ± 1.1% to 9.9% ± 1.9%, respectively.

To date, insufficient data have been acquired regarding the *in vivo* biomechanics of the MTJ due to the difficulty in attaining results during physical activity. Therefore, postmortem destructive tests are the primary source for mechanical properties of the MTJ. Several studies^27,41^ claim that the mechanical properties of the MTJ are that as displayed in Table I.

**TABLE I.** Reported mechanical properties of the myotendinous junction and its constituents.

	E (MPa)	UTS (MPa)	SAF (%)
Native MTJ	0.2789 ± 0.1509	0.1478 ± 0.016 31	122.4 ± 19.18
Muscle	0.005–2.8	⋯	⋯
Tendon	500–1850	52 –120	5–16

Upon further investigation, many of these references are obtained from studies conducted on not only different tissues but also different species. In fact, analysis of the original data referenced in these reviews provides greater insight into the variability of myotendinous tissue (see Table II). Here, ^*^ represents the corresponding value presumed to be identified in summary Table I.

**TABLE II.** Analysis of references cited by several reviews elucidating the mechanical properties of adult human MTJ. ^*^ Indicates a possible origin source for the above-mentioned summary table (Table I).

Species	Tissue	E (units defined below)	UTS (MPa)	SAF (%)	Reference
Human	Various wrist tendon	438.1 ± 93.7–726.1 ± 73.5 MPa	51.6 ± 9.3^*^–74.0 ± 13.5	11.4 ± 1.0–16.6 ± 1.7^*^	37
Human	AT	816 ± 218–822 ± 211 MPa	71 ± 17–86 ± 24	7.5 ± 1.1–9.9 ± 1.9	40
Rabbit	Patellar	549 ± 13–1390 ± 53 MPa	57.1 ± 2.5	5.3 ± 0.2^*^–14.1 ± 0.6	42
Rabbit	Patellar	955 ± 97–1855 ± 77^*^ MPa	⋯	⋯	43
Human	Patellar	504 ± 222^*^–660 ± 266 MPa	53.6 ± 10.0–64.7 ± 15.0	14 ± 6–15 ± 5	44
Rabbit	Achilles	281 ± 104.6–618 ± 87.0 MPa	23.9 ± 3.8–67.3 ± 4.2	15.7 ± 2.9^*^−16.3 ± 2.7^*^	45
Pig diaphragm	MTJ	0.2789 ± 0.1509^*^ MPa	0.1478 ± 0.016 31^*^	122.4 ± 19.18^*^	41
Human	PT	643.1 ± 53.0 MPa	68.5 ± 6.0	13.5 ± 0.7	46
Canine	PT	457 ± 98.0 MPa	122 ± 25.6	32.3 ± 7.8	47
MDX	Muscle	18 ± 6 kPa	⋯	⋯	48
C57	Muscle	12 ± 4 kPa	⋯	⋯	48
Murine	Muscle	11.5 ± 1.3–45.3 ± 4.0 kPa	⋯	⋯	49
Rabbit	TA muscle	1.75 ± 1.18–2.79 ± 0.67 kPa	⋯	⋯	50
Human	Gracilis tendon	612.8 ± 40.6 MPa	111.5 ± 4.0	33.9 ± 1.5	51
Human	Patellar	305.5 ± 59.0 MPa	58.3 ± 6.1	35.1 ± 4.4	51
Human	Patellar	307 ± 17 MPa	43.7 ± 3.9	23.4 ± 1.4	52

Comparing the original data to the referenced values (Table I), although being within similar range, this table appears to be an over-simplification of MTJ properties as it does not reveal the extent to which biomechanics vary upon location or species. Considering postmortem mechanical properties of the human MTJ constituents, Loren and Lieber studied tendons of the human wrist, observing E (at maximal tetanic tension), UTS, and SAF of 438.1 ± 93.7 to 726.1 ± 73.5 MPa, 51.6 ± 9.3 to 74.0 ± 13.5 MPa, and 11.4% ± 1.0% to 16.6% ± 1.7%, respectively.^37^ Comparatively, Johnson *et al.* studied the tensile and viscoelastic properties of the human patellar tendon, revealing an E, UTS, and SAF of 504 ± 222–660 ± 266 MPa, 53.6 ± 10–64.7 ± 15 MPa, and 14% ± 6%–15% ± 5%, respectively.^44^

Hanson *et al.* determined E, UTS, and SAF of the human AT to be 222.8 ± 84.6–316.8 ± 110 MPa, 21.9 ± 9.9–28.1 ± 9.8 MPa, and 13.8% ± 4.4%–16.3% ± 3.5%, respectively. Comparatively, the Iliopsoas tendon gave an E, UTS, and SAF of 63.5 ± 23.6–165.3 ± 67.3 MPa, 6.8 ± 2.1–22.5 ± 7.3 MPa, and 18.3% ± 3.5%–19.7% ± 5.2%, respectively.^53^ Mabe *et al.* found E, UTS, and SAF to be 201 ± 70 MPa, 16.0 ± 7.32 MPa, and 0.15% ± 0.07%, compared to that of the quadriceps tendon 153 ± 46 MPa, 19.1 ± 5.42 MPa, and 0.16% ± 0.02%, respectively.^54^

As expected, there is a greater abundance of studies conducted on animals as opposed to humans; however, the study by Pollock *et al.* revealed that the elastic properties of tendons do not vary significantly from animals of different body mass.^55^ These findings may explain how different tendons in different species consistently have properties within or close to the range found within humans.^31,42,43,45,55–66^ Similarly, the data on the tensile properties of the skeletal muscles in humans is scarce compared to that of other species.^48–50,67^

Focusing on living human mechanical properties, Maganaris *et al.* stimulated the human tibialis anterior (TA) muscle using conductive aluminum pads, enabling ultrasonography and magnetic resonance imaging of the connecting AT.^68^ By stimulating isometric loads, a maximum stress, strain, and stiffness of 25 MPa, 2.5%, and 1.2 GPa were found. Conversely, Kongsgaard *et al.* used a calf raising apparatus to study the AT revealing a max stress, strain, and modulus of 29 ± 3MPa, 4.2% ± 1.1%, and 2.0 ± 0.4 GPa, respectively.^69^ Furthermore, the Achillies MTJ averaged a proximal displacement of 7.1 ± 0.9 mm. A summary of living human mechanical properties of MTJ constituents under load can be seen below in Table III.

**TABLE III.** Mechanical properties of living, *in vivo* adult human myotendinous tissues.

Tissue	Stimuli	Analysis	E (Units defined below)	Max stress (MPa)	Max strain (%)	Reference
Free Achilles tendon	Isometric plantarflexion ramp contractions	Ultrasonography	2.0 ± 0.4–1.9 ± 0.5 GPa	29 ± 3–31 ± 4	4.2 ± 1.1–4.5 ± 1.4	69
		Ultrasonography, electromyography	788 ± 181 MPa	36.5 ± 4.6	8.0 ± 1.2	70
	Cyclic isometric contractions	MRI			2.8–4.7	71
Achilles tendon	Isometric plantar flexion	Ultrasound			5.1 ± 1.1	72
	Rest	SUE and MyotonPRO	363.38 ± 54.11 kPa			73
Surae aponeurosis and Achilles tendon	Isometric plantarflexion ramp contractions	Ultrasonography	1048–1474 MPa	41.6	4.4–5.6	74
Gastrocnemius aponeurosis (distal)	Isometric plantarflexion ramp contractions	Ultrasonography, electromyography			1.4 ± 0.4	70
Gastrocnemius tendon	Isometric plantarflexion contraction	Ultrasonography	1.16 ± 0.15 GPa	32.4 ± 2.3	4.9 ± 1	75
Gastrocnemius tendon	Isometric plantarflexion contraction	Ultrasonography	1.16 ± 0.15 GPa	32.4 ± 2.3	4.9 ± 1	75
Tibialis anterior tendon	Electrical stimulation	Ultrasonography, MRI	1·2 ± 0·15 GPa	25 ± 2·5	2·5 ± 0·4	68
Patellar tendon	Isometric knee extension	Ultrasonography	1.5 ± 0.4–1.8 ± 0.6 GPa	34 ± 8–53 ± 13	5.3 ± 0.7–5.8 ± 1.0	76
Gastrocnemius (medial)		SUE and MyotonPRO	22.59 ± 3.31 kPa			73
Gastrocnemius (lateral)			23.56 ± 4.08 kPa			73
Tibialis anterior	Rest and knee flexion	Ultrasound shear wave imaging	40.60 ± 1.00–258.10 ± 15.00 kPa			77
Gastrocnemius (medial)			16.50 ± 1.00–225.40 ± 41.00 kPa			77
Soleus			14.50 ± 2.00 to 55.00 ± 5.00 kPa			77

Apart from stimuli type and analysis method, simulation protocol, subject age, and sex, the definition of free tendon plays an important role in comparable outcomes. For example, previous methods have estimated Achilles tendon mechanical properties from measurements of distal medial gastrocnemius MTJ displacement in relation to an external marker.^69^ This method, however, does not account for elongation or contraction of the tendon–aponeurosis structure and, therefore, the results vary. It is concluded that heterogeneous analysis methods and tissue types produce inconsistent outcomes while considering the biomechanics of myotendinous tissues.

### Injuries and current interventions

Despite the adaptable and complex inner workings of the MTJ, injuries are still prevalent.^86^ Although most tears occur near the MTJ, as opposed to through it (indirect tears),^78^ the MRI reveals the resultant scar tissue is sufficient to disrupt interfacial biomechanics.^79–81^ Tears within close proximity to the MTJ generally occur between the cell membrane and lamina densa of the basement membrane.^35^

These injuries mainly arise from a passively overstressed tissue, or fast eccentric contractions, where the skeletal muscle contracts while lengthening.^82^ In fact, eccentric forces greater than 20% of the average maximum isometric force are sufficient to induce rupture.^29^

In skeletal muscles, it is widely accepted that either disrupted sarcomeres, or alternatively, damage to the excitation–contraction coupling system represents the immediate signs of injury.^83^ Furthermore, sarcomeres may be disturbed as a lack of homogeneity results in uneven energy absorption.^84^ Most commonly, muscles that are exposed to these contractions include, but are not limited to, the hamstring, rectus femoris, hip adductor, and calf muscles.^82^ Although a universal grading system is yet to be agreed upon, a classification system compromising four grades are identified by location (distal, middle, and proximal), severity, and symptoms. These grades are as follows:^85–90^

• Grade 0: edema or fluid adjacent to an intact tendon/aponeurosis/epimysium without myofibril detachment.
• Grade 1: myofibril detachment without tendon/aponeurosis/epimysium change.
• Grade 2: myofibril detachment with adjacent tendon/aponeurosis/epimysium increased signal, delamination, or defect but no retraction.
• Grade 3: myofibril detachment with adjacent tendon retraction.

Grade 1 and 2 tears may be treated with non-steroidal anti-inflammatory drugs (NSAIDS), protection, rest, ice, compression, and elevation (PRICE) protocol,^91^ and physiotherapy where a full recovery is expected, whereas grade 3 tears require surgical intervention.^92^ Currently, suturing of allografts and xenografts are the only available clinical option. A review of current suturing approaches for the MTJ may be found here.^93^ Although this generally allows for the re-attachment of the torn muscle–tendon unit, significant scar tissue results in compromised biomechanics and increased likelihood of reinjury.^93^ In cases where the interface is completely severed, suture may not allow the possibility to re-join the MTJ. Since most myotendinous injuries are untreated, surgical outcomes are not well documented.^92,94^

Furthermore, if surgery is delayed, muscular atrophy may make it impossible to re-attach. Although experimental pharmacologic agents are still being explored,^95^ suture-based approaches remain the predominant surgical intervention for repair of a grade 3 MTJ injury. Suture-based approaches remain the predominant surgical intervention for repair of a grade 3 MTJ injury.

## TISSUE ENGINEERING

Tissue engineering (TE) approaches provide great hope to assist the natural repair of musculoskeletal diseases. Recently, a trend has been developing around the hypothesis that tissue-engineered scaffolds that mimic embryonic developmental forces may dictate the differentiation of embedded cells down a specific lineage more effectively. These approaches, however, remain undeveloped and insufficient due to the limited understanding of tissue interactions during embryogenesis. Extracellular forces that contribute to embryonic development may, however, be applied to tissue-engineered scaffolds in hopes of recreating developmental-specific microenvironments. For example, Kardon *et al.* revealed that in the avian hind limb, the initial morphogenetic events, formation of tendon primordia, and initial differentiation of myogenic precursors occur autonomously with respect to one another.^96^ This effect was further iterated by Kieny *et al.* who studied the development of an embryonic chick wing. Here, they concluded that tendons start to develop autonomously from the muscle bulks, but for their maintenance and further development they require connexion to a muscle belly.^97^ Although being imperative to interfacial tissue development, *in utero* relationships remain heavily unexplored, therefore, tissue engineering advancements are predominantly governed by the understanding of individual tissues as opposed to the relationship between the two. Thus, the pillars of tissue engineering (cells, growth factors, and scaffolds) must be adapted to house complementary environments for both skeletal muscles and tendons prior to *in vivo* transplantation. Few attempts have been made to engineer the MTJ, some of which utilize scaffolds to provide initial mechanical support and disparity between skeletal muscles and tendons. Not only do scaffolds provide mechanical support but they also pre-define tissue geometry and replicate the ECM, thus allowing for provisional cellular adherence, migration,^98^ proliferation, and differentiation.^99^ To achieve desired outcomes, the following five parameters should be considered and manipulated in such a way that provokes intended biological responses:^100^

1. Biodegradability
2. Biocompatibility
3. Engineered scaffold architecture
4. Surface properties
5. Mechanical properties

Further increasing the complexity, these factors are all time-dependent, making the ideal scaffold challenging to fabricate. Scaffolds for individual tissues will not be reviewed here, as they have been elsewhere.^101^ Instead, heterogeneous structures able to mimic dissimilar tissue properties will be analyzed.

### Heterogeneous scaffolds for interfacial tissue engineering

Although planar homogeneous scaffolds are most often reported in the literature, they are incapable of producing spatially altered mechanical properties.^102^ Despite this, architectural adaptations, such as the transition from linear to crimped fibers, may be introduced to make tissue-specific scaffolds. For example, Hochleitner *et al.* used melt electro-writing below the critical translation speed to obtain sinusoidal polymer fibers.^103^ Under tensile loading, these fibers, although homogeneous, exhibited a toe region resembling the uncrimping of collagen fibrils in tendinous tissue. Producing a similar structure, Wu *et al.* fabricated a tendon-bioinspired wavy scaffold via electrospinning, displaying a tensile toe region and UTS consistent with that of the native tendon. Expectedly, however, the tensile modulus within this toe region fell below that of the native tendon, indicating optimization is required.^104^ In general, electrospun fibers maintain high surface area–volume ratios that mimic the natural ECM nanostructure. One disadvantage of electrospun fibers, however, are their ability to maintain sufficient mechanical properties, particularly for tendons, and, therefore, will only be discussed in hybrid scaffold situations. For example, Sahoo *et al.* electrospun poly (lactic-co-glycolic acid) (PLGA) nanofibers onto the surface of a knitted PLGA scaffold,^105^ resulting in increased proliferation, cellular function, failure load, elastic stiffness, and toe stiffness, whereas cell attachment remained comparable.

**FIG. 3.**
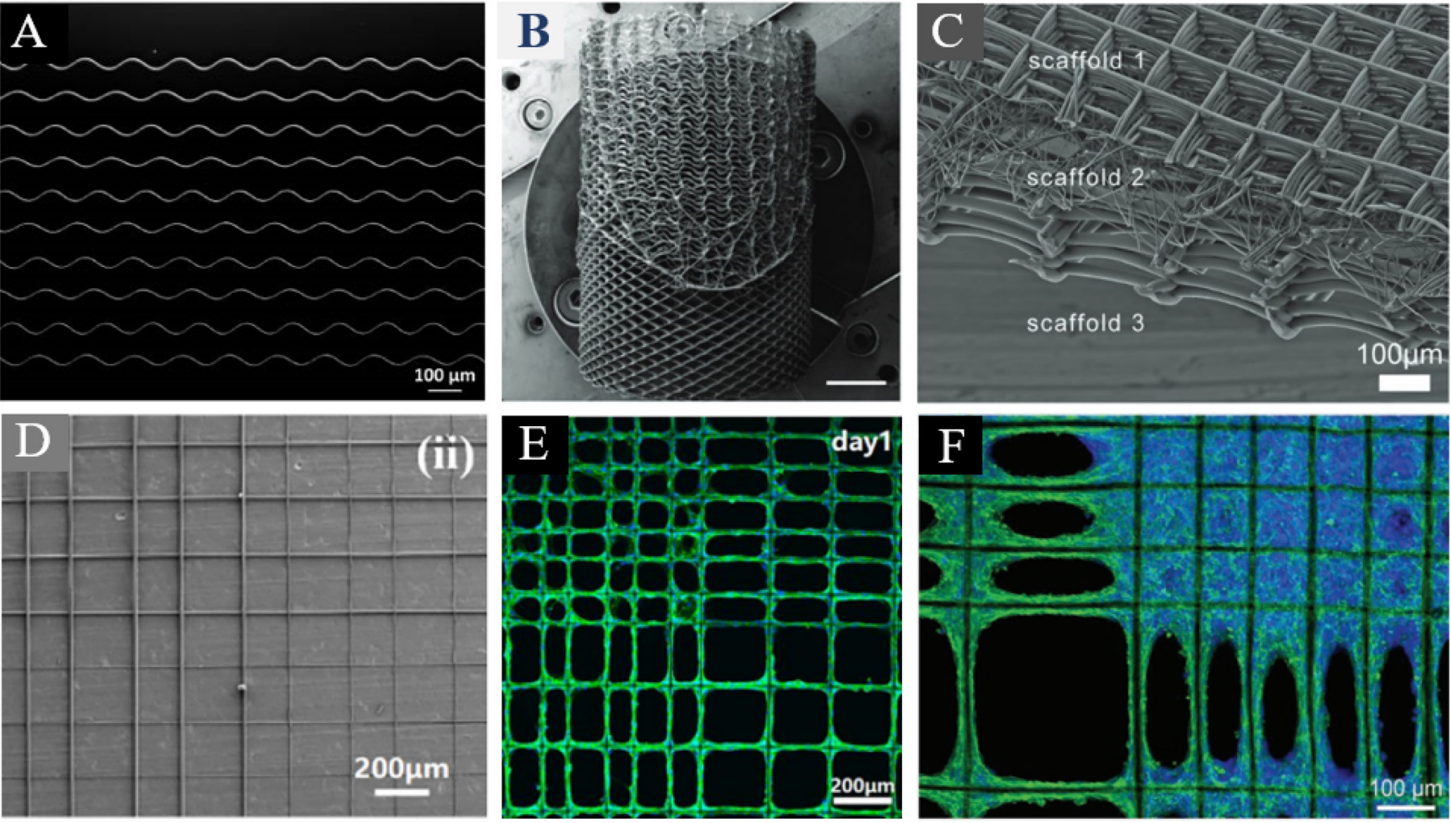
Heterogeneous scaffolds in tissue engineering (a) Fabrication of different patterns by changes in collector speed at and below CTS reproduced with permission from Hochleitner *et al.*, Bionanomaterials **17**(3), 159–171 (2016). Copyright 2016 Authors, licensed under a creative commons attribution (CC BY) license.^109^ (b) Spatially heterogeneous tubular scaffolds for *in situ* heart valve tissue engineering using melt electro-writing, reproduced with permissions from Saidy *et al.*, Adv. Funct. Mater. **32**(21), 2110716 (2022). Copyright 2022 John Wiley and Sons License.^106^ (c) Dimension-based multiphasic scaffold, reproduced with permission from Hrynevich *et al.*, Small **14**(22), e1800232 (2018). Copyright 2018 John Wiley and Sons.^110^ (d) Quadrant intersection between varying fiber diameter and pore size, reproduced with permission from Xie *et al.*, “Mater. Des. **181**, 108092 (2019). Copyright 2019 Authors, licensed under a creative commons attribution (CC BY) license.^107^ (e) HUVECs in the scaffold with pore sizes of 100 and 200 *μ*m on Day7, reproduced from reproduced with permission from Xie *et al.*, “Mater. Des. **181**, 108092 (2019). Copyright 2019 Authors, licensed under a creative commons attribution (CC BY) license.^107^ (f) Cells' reaction to various grid sizes, reproduced with permission from Nguyen *et al.*, Mater. Sci. Eng., C **103**, 109785 (2019). Copyright 2019 Elsevier.^108^

Heterogeneous scaffolds are a promising approach for mimicking the mechanical impedance seen at interfacial tissues. Melt electro-writing (MEW)-controlled deposition style presents a unique approach to achieving these mechanical and architectural variances. As practically all transplantable tissues require a 3D geometry, a cylindrical collector is often used for target tissues, such as heart valves, tendon, ligament, and skeletal muscle. For example, Saidy *et al.* printed polycaprolactone (PCL) onto a 22 mm diameter cylinder for heart valve tissue-engineered scaffolds,^106^ creating discrete and controlled architectural changes as diamond-shaped pores turned rectangular [Fig. 3(b)]. Although not having undergone appropriate tensile testing, compression testing revealed that, as expected, variations in tubular morphology had a direct impact on mechanical properties.

Steering away from biomechanics, changes in pore sizes have been shown to dictate cell attachment, alignment, and growth rate. For example, Xie *et al's* Melt electro-wrote high-resolution PCL scaffolds with four distinct zones segmented by pore size.^107^ Here, the proliferation rate of bone marrow-derived stem cells (BMSCs) and human umbilical vein endothelial cells (HUVECs) in small pores was determined to be three times faster than in larger pores, with changes in fiber diameter dictating the spread of cells, attachment, and alignment [Fig. 3(e)]. Similarly, Nguyen *et al.* seeded NIH-3T3 cells on MEW PCL scaffolds [Fig. 3(f)], confirming that cell growth is considerably influenced by lattice structure grid size, enabling the control of spatially attached cell populations.^108^

### Engineering the myotendinous junction

**FIG. 4.**
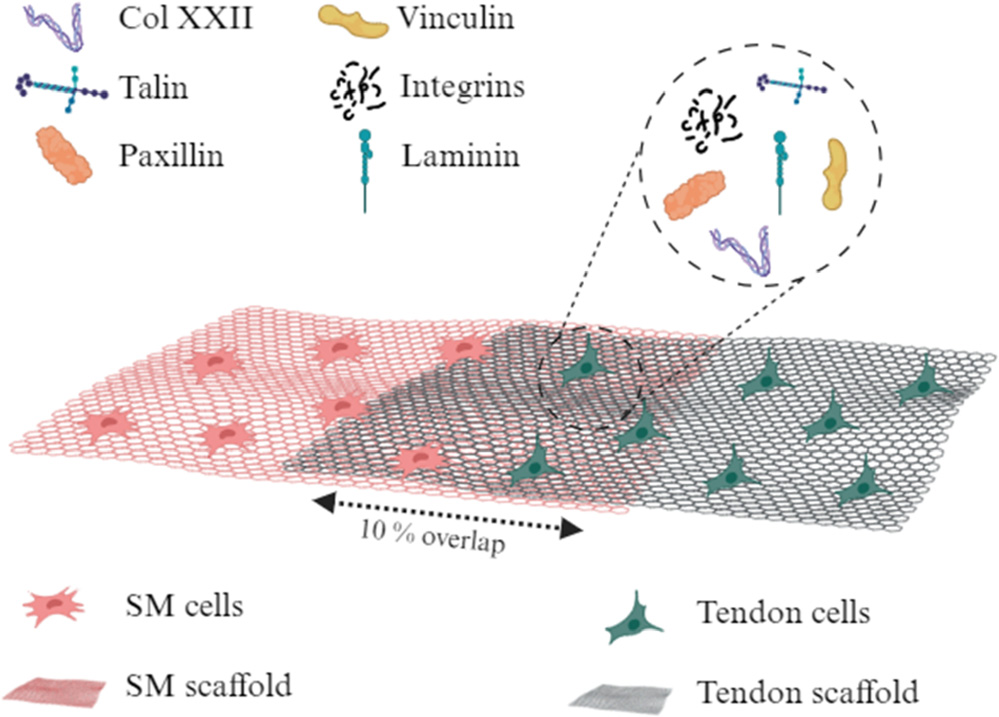
Common structural design of engineered myotendinous junction. Here, cells and scaffolds are spatially deposited, in which the 10% interfacial region signifies the main area for analysis of specific MTJ markers. Created with BioRender.com.

To date, advances in tissue engineering the MTJ rely extensively on the knowledge gained from individual tissue types. Engineering strategies can be segregated into scaffold-based and scaffold-free approaches, depending on their intended function. Scaffold-based approaches provide greater promise in mimicking the translational stiffness between skeletal muscles and tendons. Here, individual scaffolds are engineered for skeletal muscles and tendons, where an overlap defines the interface (Fig. 4).

For example, Ladd *et al.* used a 10% overlap of co-electrospun PCL and poly(L-lactide) (PLLA) fibers, creating a mechanical disparity between muscle and tendon, respectively.^41^ This unique fabrication technique produced a continuous structure, which subsequently underwent tensile loading. As targeted, the PCL side (representing SM) produced the lowest stiffness and UTS of 4.490 ± 1.604 and 1.069 ± 0.2713 MPa, whereas PLLA (representing tendon) had the highest moduli and UTS of 27.62 ± 6.063 and 3.741 ± 0.8486 MPa, respectively. The central region (representing the MTJ) gave an E and UTS of 20.06 ± 7.773 and 2.384 ± 0.5987 MPa. Conclusively, the whole composite structure produced an E and UTS of 7.339 ± 2.131 and 0.5058 ± 0.2130 MPa, respectively, and could last up to 100 cycles during cyclic loading. Subsequent embedding of C2C12 and NIH/3T3 cells into evenly distributed bovine collagen I provided no reduction in cell viability and accommodated attachment, survival, and differentiation of myoblasts into myotubes. These traits, however, did not progress into evidence of cell re-organization at the interface.

**FIG. 5.**
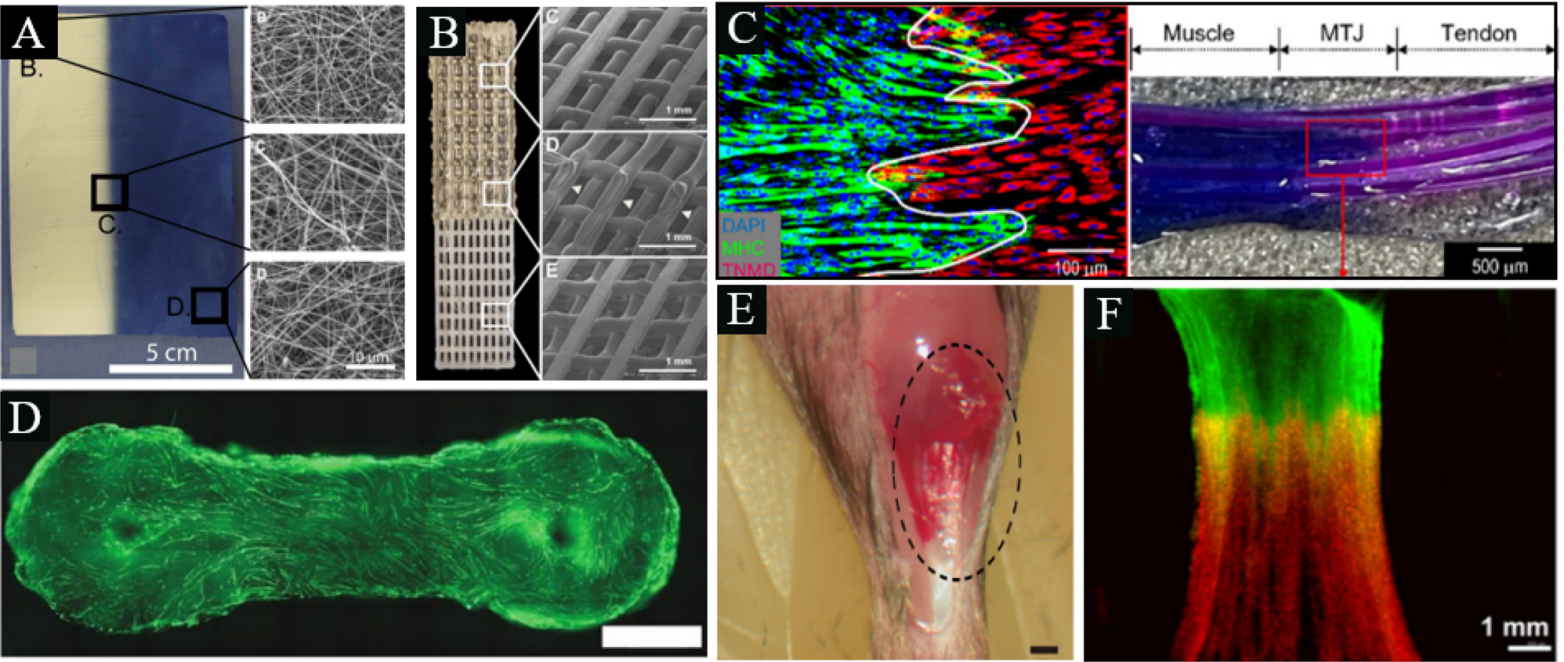
Current attempts at engineering the myotendinous junction (a) Co-electrospun triphasic scaffold compromising PCL and PLLA synthetic polymers, reproduced with permissions from Ladd *et al.*, Biomaterials **32**(6), 1549–1559 (2011). Copyright 2011 Elsevier.^41^ (b) 3D bio-printed scaffold with PCL and PU, reproduced with permission from Merceron *et al.*, Biofabrication **7**(3), 035003 (2015). Copyright 2015 IOP Publishing Ltd., through Clearance Center.^111^ (c) A bio-printed complex tissue model for myotendinous junction with biochemical and biophysical cues, reproduced with permission from W. J. Kim and G. H. Kim, Bioeng. Transl. Med. **7**(3), e10321 (2022). Copyright 2022 Authors, licensed under Creative Commons Attribution (CC BY) license.^113^ (d) A standard dumbbell-shaped muscle model, reproduced with permissions from Laternser *et al.*, SLAS Technol. **23**(6), 599–613 (2018). Copyright 2018 Elsevier.^112^ (e) Macroscopic and schematic images of procedures used in MTJ complete rupture model preparation and sheet-pellet transplantation, reproduced with permission from Hashimoto *et al.*, PeerJ **4**, e2231 (2016). Copyright 2016 Authors, licensed under a Creative Commons Attribution (CC BY) license.^116^ (f) *In vivo* culture of a 3D bio-printed MTJ construct, reproduced with permission from Merceron *et al.*, Biofabrication **7**(3), 035003 (2015). Copyright 2015 IOP Publishing Ltd,, through Clearance Center.^111^

Similarly, Merceron *et al.* also used spatial deposition of synthetic polymers with a 10% overlap to create an interfacial region.^111^ Here, bioprinting was utilized to deposit polyurethane (PU) with C2C12 cells (representing muscle), and PCL with NIH/3T3 cells (representing tendon), in which the deposition of cells had a negligible effect on viability. Mechanical characterization of the composite structure displayed a yield strain of 300%, which was expectedly driven by the superior elasticity of PU. Elastic modulus of PU, PCL, and the interface was 0.39 ± 0.005, 46.67 ± 2.67, and 1.03 ± 0.14 MPa, respectively, which, compared to *Ladd *et al.**, were mechanically inferior. On the muscle side, C2C12 cells expressed both desmin and myosin heavy chain (MHC), aligned along a unilateral plane, and began to show multinucleation, indicating their differentiation into myotubes. On the tendon side, cells began to secrete collagen I, producing a distinct interfacial region (Fig. 5). Focusing on the junction, upregulation of MTJ-associated genes, such as pax, talin 1 (tln1), vinculin, integrin β1, laminin α1, and laminin α2, were prevalent.

Yet another example of bioprinting's efficacy in creating spatially deposited co-cultures was described by Laternser *et al.*^112^ Here, a dumbbell-shaped construct was deposited in between two posts, where primary human skeletal muscle-derived cells were printed between five successive layers of gelatin methacrylate (GelMA), and tenocytes between a GelMA/polyethylene glycol dimethacrylate (PEGDMA)-based ink. Interestingly, tenocytes were printed around posts, whereas skeletal muscle-derived cells were printed in between, with a gap of 3 mm to create a clear border, maintaining a >95% cell viability. Myoblasts formed aligned areas and were able to attach to the tendinous tissue; however, after 1–2 days, the structure tore at one of the bioink boarders. The successful attachment of myoblasts to tenocytes indicated the formation of MTJ-like structures, possibly resultant from the developed tension between posts.

Another form of mechanical stimulation for engineering the MTJ was investigated by Kim *et al.*, who used a combination of biochemical and biophysical cues.^113^ One culture analyzed cell-laden human adipose-derived stem cells (hASC) in bioinks including collagen, muscle-derived ECM (mECM), and tendon-derived ECM (tECM)), in which flow-induced alignment of cells in bioinks was achieved by controlling (i) the collagen concentration in the core and (ii) the flow rates in the core and sheath. Using the idealized flow rates for optimized cellular alignment, 14 days post culture, paxillin, integrin, fibronectin, and talin were found in the MTJ region, suggesting MTJ formation. Furthermore, upregulated expression of integrin β1- and MTJ-associated genes [Pax, Tln1, thrombospondin 1 (Thbs1), collagen type 1 (Col1a1), laminin alpha 1 (Lama1), myosin heavy chain 2 (Myh2), and Scx] was identified, leading to the conclusion that MTJ formation can be clearly affected by the physical interfacing shape between muscle and tendon cells.

Scaffold-free approaches hold promise in circumventing issues associated with scaffold fabrication, degradation, and reduced biologically active volume. Larkin *et al.* evaluated the co-culture of skeletal muscle satellite cells and fibroblasts obtained from pregnant Fischer 344 rats based on their ability to create MTJs.^114^ One week of monolayer culture enabled the muscle and tendon cells to roll up into 3-D constructs around tendon tails, in which a highly aligned interface of collagen fibrils and myotubes formed. Furthermore, although less concentrated than found in adult samples, both paxillin and talin were localized at the interface. Proceeding to tensile testing, an average E of 37.2 ± 10.3 kPa (for muscle construct) was revealed, being comparable to one quarter the passive stiffness reported for young adult rat soleus muscle. Conclusively, Larkin *et al.* successfully engineered constructs resembling neonatal MTJs using a cell approach, greatly expanding the potential to control the phenotype of skeletal muscles in culture.^85^

Continuing from this, Kostrominova *et al.* adopted the same method to engineer self-organizing tendon (SOT) constructs.^115^ Briefly, once myocytes fused to form spontaneously contracting multinucleated myotubes, SOT constructs were pinned onto the muscle cell monolayer, allowing the construct to roll up around the anchors. At the early stages of culture, MTJ-like structures were represented by sub-sarcolemma densities, which later developed into well-defined folding of the plasma membrane. These plasma membranes were surrounded by type I collagen with the characteristic striation pattern.

Several other studies regarding engineered MTJ constructs have been summarized in Table IV and may be visualized in Fig. 5 below.

**TABLE IV.** Current biomaterial and biomaterial-free approaches to engineering the myotendinous junction in three-dimensions.

Scaffold	Materials	Fabrication	Cells	Location	Findings	Reference
Tri-phasic	Collagen/PCL/PLLA	Electrospinning	C2C12/NIH3T3	Vitro	• E: 4.49 (1.604), 27.62 (6.063), and 20.06 (7.773) MPa for muscle, tendon, and junction, respectively. • No indication of MTJ formation.	41
Tri-phasic	Hydrogel/PU/PCL	Bio-printing	C2C12/NIH3T3	Vitro	• E = 0.39 (0.05), 46.67 (2.67), and 1.03 (0.14) MPa for muscle, tendon, and junction, respectively. • Upregulation MTJ-associated genes paxillin, talin, vinculin, integrin β1, laminin α1, and laminin α2.	111
Mono-phasic	Polystyrene	Bio-printing	C2C12/ C3H10T1/2	Vitro	• Functionalizing oriented sub-micron fibers with printed GFs provides instructive cues to spatially control cell fate and alignment to mimic native tissue organization.	117
5 layers	GelMA/PEGDMA	Bio-printing	Rat tenocytes/human myoblasts	Vitro	• Attachment of myoblasts to tenocytes.	112
Tri-phasic	Porcine m-dECMs + fibronectin/porcine t-dECMs/ collagen I (throughout)	Bio-printing	hASCs	Vitro	• E = 40 ± 5 kPa. • Upregulated gene expression integrin β1 and MTJ-associated genes. • Paxillin, integrin, fibronectin, and talin expression. • Attachment of aligned actin filaments and collagen fibers.	113
Triphasic	Female dog dECM derived from gastrocnemius muscle and AT	⋯	⋯	Vivo	• Contractile function reached 48.4% compared to that of uninjured MTJ. • Responsive, vascularized, and innervated MTJ within 6 months of implantation.	118
⋯	SOT constructs	⋯	Fibroblasts/satellite cells	Vitro	• 14–17 days of cocultured resulted in an interface of collagen fibrils and myotubes orientated along the longitudinal axis. • Interface resembles neonatal MTJ. • Paxillin and talin concentration at junction. • E = 37.2 ± 10.3 kPa. • Failure occurred on muscle side of interface.	114
⋯	SOT constructs	⋯	Fibroblasts/satellite cells	Vitro	• Tapered myofibers integrated into collagen-rich matrix at the interface. • Interface resembles neonatal MTJ. • Some interfaces revealed folding of the plasma membrane with surrounding extracellular type 1 collagen and characteristic striation patterns.	115
Bi/triphasic	Collagen, agarose, and hydroxyapatite	⋯	Dermal fibroblasts and Sket.4u cells	Vitro	• Increased expression of tenomodulin and αSMA. • Aligned tendon and muscle cells. • Stiffness gradient achieved from muscle hydrogel (20 kPa) to tendon (140 kPa).	119
Biphasic	Type 1 collagen	⋯	Skeletal muscle cells	Vitro	• Muscle fiber ends showed invaginations of the sarcolemma. • Actin filaments from the last sarcomere terminated at the end of muscle fibers. • Basal lamina observed. • Highly contractile multinucleated muscle fibers.	120
Monophasic	Skeletal muscle-derived mesenchymal stem cells (SM-d-SCs) sheet pellets	⋯	⋯	Vivo	• Favorable results in the reconstruction and/or reconnection of ruptured muscles and tendons. • Engrafted green fluorescent protein (GFP)+ fibroblast-like cells migrated to bridge skeletal muscles and tendons. • 36% recovery in the short-term group, and 49% in the long-term group.	116
⋯	Type 1 collagen and tECM	⋯	C2C12	Vitro	• Loading upregulated Myh1, Myh2, and Myh4 in tECM. • Pax and collagen XXII expression increased in tECM and accentuated by mechanical stimulation.	121
-	tECM and mECM	⋯	C2C12 and tendon fibroblasts	Vitro	• 50%–70% paxillin increase in ECM hydrogels compared to type 1 collagen hydrogel. • ECM-conditioned media does not promote paxillin. • Collagen XXII present in tissue-derived ECM, but to a lesser extent.	122

Currently, the task of engineering interfacial tissues, especially that of the MTJ, reveals the lack of understanding in several other domains. For example, knowledge about how tissue-specific cells interact with the microenvironment and ECM of opposing tissues is scarce. Gaffney *et al.* decellularized the porcine AT and gastrocnemius muscle to form tissue-specific prehydrogel digests. Seeding of C2C12 myoblasts and tendon fibroblasts in their opposing ECM hydrogel revealed a 50%–70% increase in paxillin expression, and collagen XXII to a lesser extent.^122^ Most recently, Gaffney *et al's* continuation of this research represents the only mechanically stimulated study focused on engineering a myotendinous construct.^121^ Here, C2C12 cells were seeded in either type 1 collagen or tendon-derived extracellular matrix (tECM) and conditioned in a customed designed bioreactor. Cyclic tensile exposure of 10% strain, for 10 800 cycles at 1 Hz (3 h per day) resulted in the upregulation of myosin heavy chains (Myh1, Myh2, and Myh4), Pax, and type XXII collagen. Interestingly, tECM consistently produced greater upregulation of these factors compared to that of type 1 collagen, possibly due to the presence of tendon-specific ECM factors. Furthermore, transmission of uniaxial loads from scaffolds to cells is heavily influenced by hydrogel stiffness, as is actin–myosin striation. In fact, hydrogel mechanical properties have become increasingly influential in dynamic cultures and must, therefore, be considered an imperative aspect of interfacial tissue engineering. As previously stated, different hydrogels are commonly used in MTJ constructs; however, evolving to dynamic cultures involving uniaxial strain introduces more variables, such as tensile modulus, flexibility, and fatigue. Engler *et al.* found an optimal gel modulus of 12 kPa for skeletal muscle constructs,^48^ within the range of native skeletal muscle. Decellularized MTJ (D-MTJ) has only been reported once in the literature by *Zhao *et al.**, who seeded muscle satellite cells into D-MTJ derived from porcine AT MTJ. This study failed to investigate the coculture of tenocyte and myoblasts; however, it successfully characterized the mechanical properties of decellularized MTJ.^62^ Considering human cells, Tsuchiya *et al.* cocultured semitendinosus and gracilis tendon–muscle tissues, revealing three main findings: (i) human-derived tenocytes do not enhance proliferation of myoblasts, (ii) human-derived tenocytes enhance myotube formation, and (iii) human-derived myoblasts and tenocytes release factors important for myotube formation.^123^

In essence, the foundation of myotendinous engineering is fragile due to the limited knowledge of cellular interactions in their opposing ECMs. Therefore, it is difficult to identify the exact mechanisms responsible for the expression of interfacial characteristics, especially since this is not fully understood during *in vivo* development. The majority of data indicating the dependency of skeletal muscles and tendons during development has arisen from NULL studies, which strategically eliminate inputs, such as muscle contraction, to investigate the effect on tendon development.

### Trends and limitations

As discussed, all techniques used to engineer the MTJ have employed heterogeneous structures in combination with cocultured skeletal muscle and tendon cells. Here, the junction is represented in a slight overlap of hydrogels, cells, and scaffolds (where relevant). Although the exact mechanisms behind MTJ development are for the most part unknown, tissue engineers commonly analyze MTJ-specific gene and protein expression to determine the success of their study, such as collagen XXII. Furthermore, the existence of invaginations directly correlates with mechanical competency and is, therefore, commonly seen as an indicator of MTJ maturation. Although there has been some investigation into the response of tendon and SM cells in their opposing dECMs, this information is scarce, making it increasingly difficult to predict *in vitro* outcomes and create a targeted, standardized approach. The trend of further maturing MTJ constructs via mechanical stimuli has been identified as future steps by many authors;^41,111,124^ however, few have investigated the effect of mechanotransduction on engineered MTJ constructs.^121^ The transition to dynamic cultures must, however, be related back to the vast body of knowledge gained from individual tissues, such as skeletal muscles and tendons, respectively. As the progression from 2D to 3D cultures often produces conflicting results, it is important to only refer to 3D cultures for implantable tissue constructs.

## RECAPITULATING THE BIOPHYSICAL ENVIRONMENT

### Bioreactors

To facilitate the transition to dynamic cultures, mechanically inductive bioreactors are required. Hermetically sealed, contaminant-free bioreactors provide an environment that can mimic complex and dynamic *in vivo* environments.^125^ Bioreactors can serve three main purposes, including (i) systematic investigations to be undertaken in a controlled and reproducible manner; (ii) pre-conditioning and maturing engineered constructs prior to *in vivo* implantation; and (iii) the expansion of cells for *in vivo* transplantation.^125–127^ Pre-conditioning is of great importance as loss of cellular function is currently one of the main limitations in tissue engineering.^128^ Thus, to replicate *in vivo* interactions, bioreactors aim to control physico-chemical stimuli parameters to elicit specific cellular responses. Several bioreactor categories have been developed since the early 1980s, including rocking bed, batch stirred tank, rotating wall vessels, perfusion, and isolated expansion automated systems.^126,127,129^ Considering the myotendinous junction, stretch bioreactors are favorable; however, only few are commercially available. General bioreactor considerations revolve around the following five domains:^130^

1. Physical design/material selection
2. Mass transfer (nutrient)
3. Mechanical stimulation
4. Electrical stimulation
5. Feedback control system

Here, only two imperative domains will be described, including mass transfer and mechanical stimulation, as they have the most relevance to MTJ engineering.

### Mass transfer

*In vivo,* cells are generally within 100 *μ*m from capillary networks that provide gas and nutrient supply and waste disposal.^131^ Bioreactors must, therefore, emulate the rich and extensive vascular network of the human body via the circulation of nutrient-rich culture media. Three forms of mass transfer may occur to accommodate this in a bioreactor, including (i) convection, (ii) diffusion, and (iii) perfusion. Mass transfer in static cultures solely rely on diffusion to achieve adequate mass transfer, which, due to the diffusive penetration threshold of ∼100–200 *μ*m, commonly results in heterogeneous cell distributions.^132^ Lack of adequate perfusion is commonly visible in tissue constructs by the development of a central necrotic core surrounded by a dense layer of viable cells.^126^ As practically all transplantable tissues will exceed these dimensions and lack vasculature, mass transfer drastically restricts scalability.^125,133^ Conversely, dynamic cultures further incorporate convection via continuous media flow, and perfusion, via mass flow throughout the scaffold itself. Dynamic environments are most commonly facilitated by pumps and rotary motion; however, they may also be generated with heat exchangers, humidifiers, bubble traps, and oxygenators.

Suitable mass transfer, thus, allows culture media constituents, such as nutrients, glucose, proteins, vitamins, oxygen, and pH, to be homogeneously delivered to engineered tissue. The concentration of these constituents, however, varies with tissue growth and type and must continuously be replenished. This may be achieved via peristaltic pump systems that induce the constant supply and circulation of fresh medium. In essence, the addition of fluid flow within bioreactors drastically increases the complexity of analysis and design considerations. For example, pumping flow rate may be set to induce laminar or turbulent flow, which is further influenced by a bioreactor's architecture.^134^ Ideally, mass transfer from supplying arteries and arterioles of the perimysium at the myotendinous junction must be emulated to avoid necrosis and enable reliable tissue growth.

### Mechanical stimulation

Regarding the maturation of myotendinous structures, the implementation of mechanical forces *in vitro* appears to increase tissue maturity. To date, there is convincing evidence that physical forces are imperative for tissue development during embryogenesis.^126^ In fact, a study by *Sungsoo *et al.** displayed that stress-induced signal transduction is at least 40 times faster than growth factor-induced signal transduction while considering the human airway smooth muscle (HASM) cells.^135^ Forces assumed to contribute to this consist of hydrodynamic^125^ and hydrostatic pressure, fluid dynamics, mechanical stresses and strains, and electrical cues.^133^ To replicate one aspect of these, a dimension-based mechanical stimulation is often embedded into bioreactors.^136^

In three dimensional tissue culture, forces may be applied via three mechanisms, including (i) fluid flow, (ii) mechanical vibration, and (iii) mechanical stimulation.^137,138^ Focusing on mechanical stimulation, forces may be applied via uniaxial or biaxial tension, compression, and/or torsion depending on the native tissue environment that is being emulated. Tensile strain is the most commonly applied biophysical stimulus used to mimic the native muscle environment. While each individual muscle belly shortens during movement, the contraction of individual myofibers against two ends that are tethered to the rigid bone induces tensile strains in the tissues as they are contracting.^139^ In reported stretch bioreactors, one end is often clamped into a stationary position, whereas the other is clamped to an oscillation source, commonly connected to a stepper motor.^140^ Here, strain concentrations increase in magnitude approaching the clamps, leading to altered cellular responses which may be able to mimic interfacial tissues, such as the MTJ.

Studies demonstrate that, dependent on the exposure routine, mechanical input can (i) stimulate ECM production,^141^ (ii) improve cell/tissue organization,^136^ (iii) direct cell differentiation^142^ and alignment,^143^ and (iv) enhance targeted tissue functions.^144^ For example, by analyzing cells more susceptible to stretching, such as fibroblasts, cellular alignment along the axis of strain can be observed after just three hours of exposure.^145^ This is expected as fixation of a scaffold between two anchor points creates predictable lines of isometric strain which cells sense and respond to accordingly. This mechanically mediated internal tension is sufficient in promoting cellular alignment along the principal axis of strain,^140,146,147^ and as previously described, was used by both Laternser *et al.*^112^ and Gaffney and Laternser^121^ to engineer the MTJ.

Considering uniaxial strain exposure, little is known about which specific mechanical force(s) or regimes of application (magnitude, frequency, and duty cycle) induce specific cellular responses.^131^ Brown *et al.* showed that tissue engineering programs should be customized for axial vs limb tendons while studying Scx-GFP mice cells under a cyclic uniaxial strain.^148^ Significant consideration must, therefore, be given to determine the appropriate application of biophysical stimuli, such as static vs cyclic, continuous vs intermittent, low vs high amplitude, among others. For example, a frequency of 1 Hz is similar to the natural stride frequency, mimicking the strain cycle of muscles in locomotion.^139^ Referring back to embryonic cues however, Kodama *et al.* found that muscle-driven movements began at E14 in mice, creating a timepoint benchmark for the transition from static culture to mechanically inducive dynamic cultures.^24^ These findings have further implications to the cues dictating tendon development, suggesting tendon progenitor cells (TPCs) begin to differentiate as a function of their particular anatomical microenvironments prior to E14. This was further consolidated by Lipp *et al.*, who found muscular contraction to begin at E13.5.^149^

Adhering to this embryonic phenomenon, Foolen *et al.* applied a cyclic stretch after a static culture to study the subtle balance between contact guidance and stress concentrations in 3D.^147^ Here, cells in the core displayed alignment along the principal axis of the strain; however, cells became increasingly oblique toward the exterior due to decreasing contact guidance toward the exterior of the construct. Similar results can be seen from Boerboom *et al.*^150^ and Rubbens *et al.*^151^ Conversely, Chen *et al.* restrained cell deformation between two posts for 24 h before applying a cyclic uniaxial strain, resulting in homogeneously aligned cells from surface to core.^146^ It has been suggested that the earliest forces experienced by MTJ constituents is via the elongation of the connecting bone,^16^ in which the increasing cell-mediated tension between bioreactor posts may have mimicked this function and enhanced homogeneous cellular alignment.^146^

Cyclic loading, however, produces inconsistent results on signaling and myogenic marker expression, making it difficult to determine optimal cyclic parameters, whereas static loading largely produces consistent results. Although the exact science behind mechanotransduction is yet to be elucidated, it has been hypothesized that the strain effect on myogenesis may occur by signaling intracellular transcription factors. This further iterates that the expression of myogenic markers is a direct indicator of the progression of immature constructs to functional implants. There have been many studies conducted on the effect of mechanical strain on skeletal muscles and tendons individually; however, there have not yet been many studies specifically investigating the effects of mechanical strain at the MTJ (see Tables V and VI).

**TABLE V.** 3D tendon constructs under uniaxial strain.

Cell Type	Type	Substrate/scaffold	Mechanical parameters	Effects	Reference
Tendon-derived stem cells (TDSCs)	Cyclic	Confluent monolayer with ECM deposition (rolled up)	6% strain, 0.25 Hz, 8 h/d, 6 days	• Uniaxial > biaxial for tenogenic differentiation markers. • Compact, aligned, and connected F-actin. • Increased expression of Scleraxis, Mohawk, TNMD, and COL1A1. • Osteogenic markers also relevant.	152
hMSCs	Cyclic	Electrospun polycaprolactone fibers	Intermittent 12% strain, 0.01in/s, for 21 days	• Increased collagen and ECM production • Fiber arrangement has no discernible pattern. • Addition of cells increased elastic moduli from 30.33 ± 8.37 to 216.53 ± 55.71 MPa	141
BM-dMSCs	Cyclic	Decellularized tendon scaffolds	0%, 3%, or 5% strain at 0.33 Hz for up to 1 h daily for 11 days	• 3% strain gave best results • UTS of 17.7 ± 3.8 MPa • Elastic moduli of 119 ± 44 MPa, within 25% of native tendon value (98 ± 25 MPa) • Altered ECM composition. • Deep cellular integration • Tendon-like gene expression	153
BM-dMSCs	Cyclic	Raw silk knits	12h/day, 0.1 Hz	• Collagen synthesis increased. • Improved cellular elongation and ECM deposition along the direction of the mechanical strain. • Upregulated gene expression (collagen 1, tenascin-C, and TNMD). • Minimal differences in tensile properties	154
Fibroblasts	Cyclic	Elastomeric polyurethane (Tecoflex SG-80A)	10% strain, 0.25 Hz, 8 h/day	• Increased proliferation • Presence of ascorbic acid results in increased elastic modulus in the high strain region • Increased fibroblast DNA content • Ascorbic acid increases collagen 1 and fibronectin matrix accumulation. • Homogeneous distribution of ECM and cells.	155
TDSCs	Cyclic	Poly(L-lactide-co-ε-caprolactone)/collagen (P(LLA-CL)/Col) scaffold	2%, 4%, and 8% strain at 0.3, 0.5, and 1.0 Hz	• No effect on cell viability • 0.5 Hz and 4% strain gave produced greatest increase in proliferation • Increased collagen 1, tenascin-C expression, TNMD, and SCX	156
MSCs	Cyclic	Collagen fiber scaffold	10% strain, 1 Hz, 3 h on/3 h off, 14 days	• Increased SCX • Increased ECM deposition (collagen 1, collagen 3, and tenascin-C)	157
Fibroblasts	Cyclic	Collagen disk fiber	0.1 Hz, 0%–0.7% strain, 7 days	• Increased SCX and TNMD • Fiber nuclei deformed to more or a spindle (elongated) morphology, aligned to the long axis of the fiber. • Increased peak stress, Young's modulus, and toughness. • No change in failure strain or toe-in strain	158
Tenocytes	Cyclic	Collagen sponge	3% preload followed by 10% strain at 0.5, 1, and 2 Hz.	• Scaffold moduli of 17.5 kPa, and stiffness of 0.022 N/mm • Tenocytes and scaffold fibers promoted homogeneous alignment	159
hMSCs	Cyclic	PCL electrospun yarns	5% strain, 1 Hz, for 1 h per day	• More textured and rounded cells • No evidence of cell infiltration into the structure • Upregulation of Col1a1, Col1a2. Col3a1, tenascin-C, elastin, and fibronectin. • Increased Young's modulus and UTS.	160
BMSC's	Cyclic	Decellularized tendon slices	3% strain, 0,2 Hz, 12 h/day, 7 days	• Upregulated decorin and TNMD • No changes in UTS or stiffness • Enhanced cell infiltration deeper into the tissue • Improved ECM protein expression • Alignment along principal axis of strain	161
MSCs	Cyclic	Decellularized tendon matrix scaffolds	2% strain at 1 Hz (various regimes)	• Strain decreased cellular alignment. • Short stretching may promote cell integration. • Downregulated collagen 1A2 expression • Upregulated collagen 1A2 and 3A1, and SCX	162
MSCs	Cyclic	Electrospun PCL nanofiber yarns	4% strain at 0.5 Hz, 2 h/day for 12 days	• Promotion of TNMD and COL1 • Increased total collagen content. • Increased SCX, TNC, COL1, COL3, TNMD, and VEGFA genes	163
MSCs	Cyclic	Collagen 1 sponges	Stimulated once every 5 min for 8 h/d to a peak strain of 2.4% for 2 weeks.	• Increased collagen 1 and 3 • No significant increase in decorin, fibronectin, and GAPDH gene expression • Linear modulus increased from 0.0005 ± 0.002 to 0.02 ± 0.01 MPa • Maximum stiffness increased from 0.003 ± 0.001 to 0.005 ± 0.002 MPa	164
Fibroblasts	Cyclic	Bioartificial tendons	1 h/day at 1% elongation and 1 Hz	• Increased nuclei elongation and cytoplasmic extensions • Modulus increased from 0.49 to 1.8 MPa • UTS strength increased 2.9 fold after 7 days	165
AT	Cyclic	Achilles tendon	0.25 Hz for 8 h/day, 0%–9% for 6 days	• 3% strain results in slightly disrupted ECM structure and significant cellular morphology changes. • 6% strain circumvented the previously mentioned issues, and also had no type 3 collagen expression. • 9% strain resulted in partial tears and 45% apoptosis.	166
MSCs	Static	GelMA hydrogel yarn	15% for 7 days, with an additional 15% from day 7–14	• Higher fraction of elongated cells and spreading. • Cell alignment up to 50% in the direction of stretch.	167
MSCs	Cyclic	Human umbilical cord	2% strain, 0.5 h/day, 0.5 cycle/min, and 14 days	• Decreased cell infiltration. • Some alignment in direction of stretch, but ECM still somewhat disorganized/wavy. • Increased UTS. • Day 7 revealed upregulated collagen type 1 and 3, and tenomodulin. • Day 14 revealed downregulated collagen type 1 and 3 while elastin and SCX were upregulated.	168
TDSCs	Cyclic	P(LLA-CL)/Col	4% strain, 0.5 Hz, 2 h/day, and 14 days.	• Increase in cell number. • More uniform distribution of cells. • Col 1 and 3, decorin, tenascin C, and biglycan upregulated. • Downregulated Runx2, Col 2, and aggrecan • UTS increased from 43.18% ± 6.58% to 59.58% ± 7.81% of normal patella tendon in rabbits. • Young's modulus increased from 23.30% ± 3.83% to 51.99% ± 7.16% of normal patella tendon.	169
BM-MSCs	Cyclic	Human umbilical cords	2% strain, 1 h/day, 1 cycle/min, calibrated 1% strain during rest periods (several other variations)	• Increase in cell number. • UTS ranging from 1.06 (0.34) to 1.58 (0.35) MPa • Increased fiber alignment, although quite far from ideal scenario. • Upregulation of biglycan, tenascin C, Col 1, and Col 3. • Slow frequencies produce significant decorin downregulation.	170
Fibroblasts	Cyclic	Human flexor and extensor tendon	0.625–2.5 N, up to 8 days	• Conditioning duration has significant effect on material properties, the load magnitude does not. • Increased UTS and elastic modulus for all loading regimes.	171
MSCs	Cyclic	Braided hyaluronate elastic band	1 Hz at 0.1 N (≈10% strain)	• Increased collagen type 1 and 3, decorin, SCX, and tenascin-C. • Biochemical and mechanical stimuli was used in tandem.	172
MSCs	Cyclic	Collagen type 1 sponge	0%–2.4% strain, 1 Hz for 20 s followed by 100 s rest, for 5 h/day	• Increased type 1 collagen. • Increased linear stiffness.	173
MSCs	Cyclic	Poly(ethylene glycol)-based hydrogel	10% strain, 1 Hz, 3 h of strain followed by 3 h without	• No change in cell number or viability • Increased Col1, Col2, and tenascin C.	174
MSCs	Cyclic	Decellularized tendon scaffold	3% strain, 0.33 Hz, 1 h/day	• Increased SCX, Col1, Col3 • Decreased TNMD • Increased mechanical properties.	175
Tenocytes	Cyclic	Decellularized tendon	1.25N stretch, 1 cycle/min, in alternating 1 h periods of load and rest, over 5 days.	• UTS increased from 35.69 ± 5.62 to 71.17 ± 14.15 N. • Elastic modulus increased from 632 ± 86 to 1091 ± 169 MPa (no significant difference from freshly harvested tissue)	176
MSCs	Cyclic	Collagen sponge	2.4%, at 1 Hz, 8 h/day, with either 100 or 3000 cycles/day.	• Stiffness decreases with increase cycles/day. • Col 1, 3, and Decorin was maximal at 3000cycles/day, with a minimal value at 100cycles/day. • Fibronectin expression was maximal at 0cycles/day, and minimal at 100cycles/day.	177
BMSCs	Cyclic	Collagen sponge	15% strain, 1 Hz, and 3 days	• Increased SCX and TNC • Decreased Col1a1 expression. • Failure strain increased from 98.58% ± 8.92% to 116.40% ± 14.51%. • Young's modulus decreased from 14.17 ± 0.87 to 7.80 ± 1.72 kPa. • Increased collagen scaffold porosity.	178
MSCs	Cyclic	Collagen type 1	1% strain, 1 Hz, 30 min/day, and 7 days	• 50% increase in collagen production. • SCX initially decreased, but subsequently increased. • Wnt5a and Wnt14 downregulated. • Wnt16, Col1,3, XII, and elastin upregulated.	179
Fibroblasts	Cyclic	Collagen type 1	10% strain for 15 min, 0% strain for 15 min, and 15 min rest for 24 h	• Cells aligned parallel to force from 110° to 180° (not seen in static cultures) • Upregulated collagen I, collagen III, TNMD, and TGF‐β	143

**TABLE VI.** 3D skeletal muscle constructs under uniaxial strain.

Cell Type	Type	Substrate/scaffold	Mechanical parameters	Major findings	Reference
C2C12	Static	Fibrin	10% static strain for 6 h, followed by 18-h rest at 3% static strain. Repeated for 6 days.	• Higher degree of alignment (>85% in direction of strain, residual 15 showed a 15%–30% deviation) and preservation. • Thicker (≈ 20%) and longer (≈30%) myotubes. • Greater sarcomeric patterning.	136
Myoblasts	Cyclic	Collagen	10% stretch 3 times/minute for the first 5 min of every h, duration ranging from 5 days to 3 weeks.	• Uni-directional alignment achieved within 5 days and maintained *in vivo*. • Depolarization with KCI resulted in measurable contractile responses after 3 weeks; however, dissipated within 1 week *in vivo*.	180
Myoblasts	Static	Collagen	Sustained uniaxial tension (unspecified)	• Sustained uniaxial tension promotes myoblast fusion to form aligned multi-nucleate myotubes. • Myotube alignment parallel to strain. • Surge in both IGF-1 Ea and MGF expression on day 3 of the developing construct	181
Myoblasts	Static/cyclic	Collagen sponge	7.5%–15% static strain for 6 h.	• Increase MMP-2 expression and matrix remodeling. • Upregulation depends on amount and type of strain.	182
C2C12 myoblasts	Static	Fibrin gel	25% strain	• Cells aligned parallel to strain direction. • Cell proliferation was identical to that cell alignment. • Adjacent cells were not in contact, due to fibrin bundles restricting cellular activity to the space in between. • Increased proliferation compared to control, and 50% strain produced enhanced proliferation compared to 25% strain.	183
Muscle-derived cells (MDCs)	Static	Collagen constructs	Isometric tension (no strain)	• Aligned myotubes. • Tension increased as structure contracted, causing concave center.	184
C2C12 myoblasts	Cyclic/static	Collagen matrix	Rotation of strain increase, strain maintenance, strain release, and rest for 6 days	• IGF-IEa constitutively expressed in myoblasts and myotubes under endogenous tension. • Single ramp stretch and cyclic loading increased mechano-growth factor (MGF), whereas static strain did not.	185
C2C12 myoblasts	Cyclic	Aliphatic diisocyanate-based polyurethane (PU) fibers	(i) 5% strain at 1 Hz, (ii) 10% strain at 1 Hz, and (iii) 5% strain at 1 Hz with constant 5% pre-stretch. 1 h on, 5 h rest.	• Myotubes exhibited evidence of overstretching at both 5% and 10% strains. • Very few myoblasts fused to form myotubes on the scaffold, and those that did were short and lacked sarcomeric structures. • Overstretching resulted in a proliferation effect, as seen in response to injury. • Produced increased fraction of striated myotubes from 75% to 85%.	186
Muscle precursor cells (MPCs)	Cyclic	Acellular collagen matrices	10% strain stretched 3 times per minute for the first 5 mins of every hour for periods ranging from 5 days to 3 weeks.	• Unidirectional orientation within 5 days. • Generation of contractile response after 3 weeks. • Conditions tissue produced contractile responses with 1% and 10% specific force *in vivo*, whereas non-conditioned tissue does not.	180
MPCs	Cyclic	Porcine bladder acellular matrix scaffold	10% strain, three times per minute for the first 5 min of every hour, for one week.	• *In vivo* implantation resulted in decreased recovery time. This, however, also incorporated electrical stimulation.	187
MDCs)	Cyclic	Bladder acellular matrix scaffold	10% strain, three times per minute for the first 5 min of every hour, for 5–7 days	• Cells exhibited an elongated and aligned morphology. • TEMR-1SPD produced a significant reduction in nuclei, whereas TEMR-2SPD produced a significant increase. • Strain produced higher levels of multinucleated cells.	188
Fibroblasts	Cyclic	Collagen gel	480 dynes/h, 15-min intervals of load, rest, unload, rest for 16 h. Initially loaded with 120 Dyne force.	• High aspect ratio configurations produced dramatic alignment parallel with the axis of strain, shape control of cells, elongated, and approximately bipolar, whereas low aspect ratio configurations did not (after 16 hours). • Alignment is predominately “nose to tail” configuration. • No alignment of collagen fibrils was evident.	189
Skeletal muscle cells	Cyclic	Collagen/MATRIGEL	5%, 10%, and 15% strain. 5 stretch and relaxation cycle. Repeated 3 times with rest in between. Total duration of 30 minutes.	• Internal longitudinal tensions align cells into parallel arrays which then fused into aligned multinucleated myofiber. • Mechanical conditioning of HBAMs improved HBAM myofiber diameter and area percentage.	190
Fibroblasts	Cyclic	Polyethylene terephthalate (PET)	5%, 0.5 Hz, 1 h daily, 14 days	• GAG per DNA was statistically lower in the bioreactor as compared to the static culture. • Stretched and non-stretched studies produced a sevenfold increase in cell numbers compared to Petri-dish cultures. • Little differences seen between stretch and non-stretched specimens.	191
Cardiac progenitor cells (CPCs)	Cyclic	Decellularized human skin (d-HuSk)	10% strain, 1 Hz, for 7 days.	• Dynamic culture promoted hCPC migration toward the inner layers of the scaffolds. • Upregulated cardiac alpha actin. • CD117 and TBX3 significantly down regulated.	192
MSCs	Cyclic	Hyaluronate band with 3D fibrin hydrogel	10% strain, 1 Hz for 72 h.	• Upregulation of SCX, type 1 collagen, decorin, and tenascin-C.	193
Mesenchymal stromal cells	Cyclic	Non-crosslinked porcine-derived acellular dermal matrix	1%, 5%, and 10% strains, 0.2, 0.33, and 0.5 Hz.	• Sustained SCX and tenascin C increase. • Wnt 16 and tenomodulin time-dependent increase.	194
hADSCs	Cyclic	Spider silk with type 1 collagen	2% strain for 8 h daily at 1 Hz. 16 h rest between every stressing. 21 days duration	• Cells and silk fibers orientated and aligned toward the direction of mechanical stimulation. • Cell density decrease.	195
C2C12 Myoblasts	Cyclic	Collagen gel	3% strain, 1 Hz, 4 h/day, for 2 days	• Cell diameter decreased. • Elongated and aligned along direction of strain. • Increased differentiation and myotube size.	142
C2C12 Myoblasts	Cyclic	DegraPol microfibrous membranes	Phase C: 1 mm, 0.5 Hz (30 s rest between each pulse), 28 min rest	• Eightfold increase in myosin content.	196
C2C12 Myoblasts	Static	GelMA	0%–45% strain for 5 days	• Improved cell spreading. • Improved elongation. • Improved alignment along direction of strain.	197
C2C12, MPCs	Cyclic	Fibrin	2-day uniaxial ramp stretch (0%–2%), followed by 2%–6% dynamic stretch (3 h on, 3 h off)	• Orientation of myotubes in direction of attachment between anchor points. • Increased cross striations in MPC constructs.	144

**FIG. 6.**
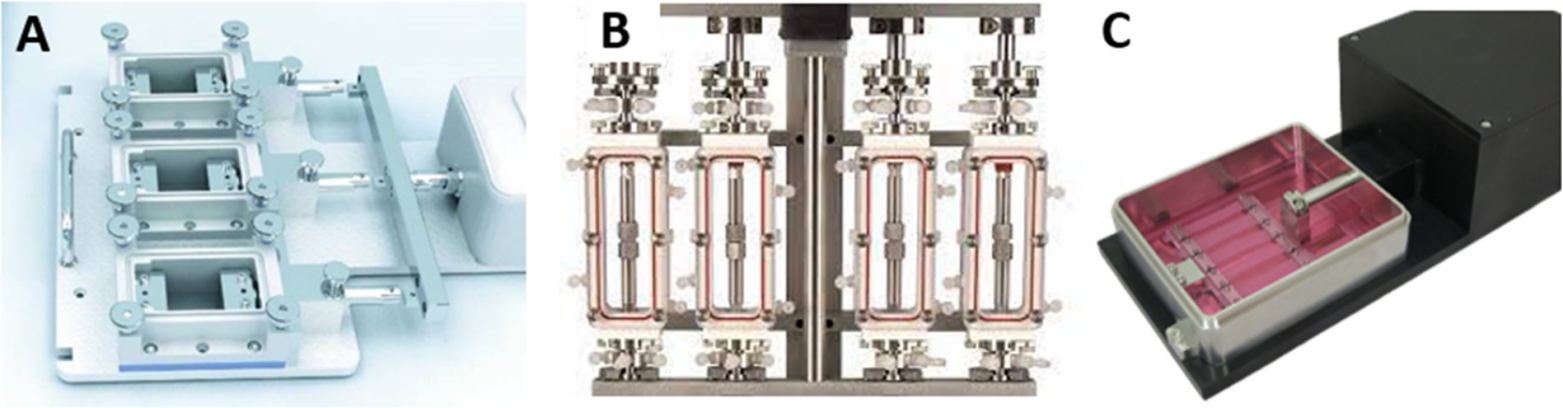
Example of commercialized bioreactors capable of applying a uniaxial strain to 3D tissue-engineered constructs. (a) Ebers TC-3 bioreactor (ceeiaragon.es). (b) Biodynamic 5270^*^ bioreactor (docplayer.net). (c) Cellscale MCT6 bioreactor (cellscale.com).

Overall, the mechanical stimulation of skeletal muscle cells promotes a higher degree of alignment in the direction of the strain,^136^ thicker and longer myotubes,^136^ greater sarcomeric patterning,^136^ promoted myoblast fusion,^181^ significantly higher MMP-2 expression,^182^ enhanced proliferation,^183^ unidirectional nose to tail orientation,^189^ generation of contractile force,^180^ elongated and aligned morphology,^188^ higher level of multinucleated cells,^190^ promoted hCPC migration,^192^ and upregulation of scleraxis, collagen I, decorin, and tenascin C^193^ while maintaining comparable cell survival.^198^ Similarly, engineered tendon constructs promoted alignment,^143,161,167^ ECM production,^157,161,179^ maturation,^160,169,178^ and expression of tenogenic markers.^143,175,178^ As depicted above, a uniaxial strain has a profound effect on various cellular activities. Further studies are reviewed by *Somers *et al.**^139^ To induce this strain, several strain-inducing bioreactors have been commercialized and are available from a number of companies (Fig. 6) including Strex/Strexcell, Electroforce, Flexcell, Biodynamic, Tissue growth technologies, Ebers, Cellscale, and Con Whitley scientific.

The vast majority of strain bioreactors have been custom designed and are primarily research focused, such as those seen in Table VII. Lab-specific bioreactors commonly incorporate a 3D-printed frame, transparent Perspex lid, stepper motor linear actuator, and a wide range of clamps to constrain the engineered tissue, similar to commercialized devices. Here, a wide range of materials, including Perspex, silicon, aluminum, polycarbonate, resin, polyurethane, Teflon, and stainless steel, are utilized, which all achieve self-proclaimed biocompatibility.^189–191,199–201^ Given this large range of materials, however, various cleaning protocols and surface treatments are mentioned. For example, to improve biocompatibility and hydrophobicity, sylgard-184 has previously been coated on the bottom of a resin-based bioreactor chamber.^202^ Similarly, basic features, such as gas vents, are common throughout, where only the most intricate designs incorporate mechanical feedback (via in line or bending beam load cells) and automated media exchange systems. Finally, there are a finite number of designs that incorporate electrical stimuli with a uniaxial strain;^158,186,200,203–205^ however, they will not be summarized here as they are not the focus of this review.

**TABLE VII.** Custom-made bioreactors capable of providing uniaxial strain on three-dimensional tissue-engineered constructs. ✓ indicates transparent at top and bottom. ✓✓ indicates transparent at top and bottom.

Stretch **source**	Separate chambers?	No of samples	Metallic free media?	Sensory feedback (mechanical)?	Automated media exchange	Media exchange ports?	Gas exchange filter?	Transparent?	Reference
Stepper motor driver	✗	Unspecified	✗	✗	✗	✗	✗	✓✓	199
Lead screw micro-stepping motor	✓	1	✗	✓	✗	✗	✓	✓✓	189
Linear actuator stepper motor	✓	6	✓	✓	✗	✓	✓	✓	190
Linear actuator stepper motor	✓	1	✗	✓	✗	✓	✓	✓✓	206
Linear actuator	✗	12	✗	✗	✓	✓	✗	✓✓	191
Micro-stepper motor	✓	1	✗	✗	✗	✗	✓	✓✓	207
Stepper motor	✓	1	✓	✗	✗	✓	✓	✓	192
Linear motor	✓	1	✓	✗	✗	✗	✗	✓✓	193
Micro-gear motor	✓	1	✓	✓	✗	✗	✓	✓	208
Motor (unspecified)	✓	1	✗	✓	✓	✓	✓	✓	194
Driving motor (unspecified)	✓	1	✗	✓	✓	✓	✗	✓✓	209
Drive motor (unspecified)	✗	5	✗	✗	✗	✗	✓	✓✓	195
Electric servomotors	✓	1	✗	✓	✓	✓	✗	✓✓	210
Linear motor	✗	10	✓	✗	✗	✗	✗	✓✓	180
Linear activator stepper motor	✓	12	✗	✗	✗	✗	✗	✓	211
Stepper motor actuator	✓	6	✗	✗	✗	✗	✗	✓✓	212
Linear actuator	✓	6	✓	✗	✗	✗	✓	✓	202
Motor-ball screw actuator	✓	6	✗	✗	✗	✗	✗	✓	166
Linear actuator stepper motor	✗	6	✓	✓	✗	✗	✗	✓	141
Linear actuator stepper motor	✓	1	✗	✓	✗	✗	✓	✓	213

## CONCLUSION

It is widely hypothesized that engineered tissues that mimic embryonic (as opposed to adult) mechanical properties will produce greater biological responses. Although producing promising results, the paucity of knowledge surrounding MTJ's embryonic development, particularly that of skeletal muscle, makes outcomes difficult to quantify. Currently, attempts to engineer the MTJ rely on heterogeneous scaffolds aimed to provide the appropriate environment for skeletal muscle and tendon tissues simultaneously. In doing so, the interfacial region may be analyzed for interface-specific genes, proteins, and architectural characteristics. To date, although favorable results have been achieved, the mechanical discrepancy and architecture complexity between skeletal muscles and tendons is yet to be mimicked. To overcome this issue, many groups identify the possibility to mature myotendinous constructs in a dynamic, strain-inducive bioreactor as a means of tissue maturation;^41,111,124^ however, few have advanced to this stage.^121^ The translation to dynamic cultures must be reliant on the established body of knowledge known about individual tissues, such as skeletal muscles and tendons separately. Here, the significant progress of engineering tissues may be merged to develop targeted strategies focused on interfacial regions such as the MTJ. Future studies are advised to direct their attention to the translation from static to strain-inducive dynamic bioreactors, where a cyclic regime following an initial static strain period shows the greatest results.

## Data Availability

Data sharing is not applicable to this article as no new data were created or analyzed in this study.
